# Sex-Associated Differences in Neurovascular Dysfunction During Ischemic Stroke

**DOI:** 10.3389/fnmol.2022.860959

**Published:** 2022-04-01

**Authors:** Tianchi Tang, Libin Hu, Yang Liu, Xiongjie Fu, Jianru Li, Feng Yan, Shenglong Cao, Gao Chen

**Affiliations:** ^1^Department of Neurosurgery, Second Affiliated Hospital, School of Medicine, Zhejiang University, Hangzhou, China; ^2^Department of Ultrasonography, Shanghai General Hospital, School of Medicine, Shanghai Jiao Tong University, Shanghai, China

**Keywords:** ischemic stroke, neurovascular unit, sex dimorphism, gonadal hormone, neuroinflammation, genetic, epigenetic

## Abstract

Neurovascular units (NVUs) are basic functional units in the central nervous system and include neurons, astrocytes and vascular compartments. Ischemic stroke triggers not only neuronal damage, but also dissonance of intercellular crosstalk within the NVU. Stroke is sexually dimorphic, but the sex-associated differences involved in stroke-induced neurovascular dysfunction are studied in a limited extend. Preclinical studies have found that in rodent models of stroke, females have less neuronal loss, stronger repairing potential of astrocytes and more stable vascular conjunction; these properties are highly related to the cerebroprotective effects of female hormones. However, in humans, these research findings may be applicable only to premenopausal stroke patients. Women who have had a stroke usually have poorer outcomes compared to men, and because stoke is age-related, hormone replacement therapy for postmenopausal women may exacerbate stroke symptoms, which contradicts the findings of most preclinical studies. This stark contrast between clinical and laboratory findings suggests that understanding of neurovascular differences between the sexes is limited. Actually, apart from gonadal hormones, differences in neuroinflammation as well as genetics and epigenetics promote the sexual dimorphism of NVU functions. In this review, we summarize the confirmed sex-associated differences in NVUs during ischemic stroke and the possible contributing mechanisms. We also describe the gap between clinical and preclinical studies in terms of sexual dimorphism.

## Introduction

Stroke, especially ischemic stroke, is a leading cause of mortality and disability worldwide (Mozaffarian et al., 2015), and in China, stroke is the third leading cause of death (Wang Y. J. et al., 2020). Sexes differently respond to stroke (Manwani and McCullough, 2019), and researches on stroke should be designed on the basis of the different sexes (Albers et al., 2011). The concept of neurovascular units (NVUs) may enable a better systematic and comprehensive understanding of ischemic brain injury.

Neurovascular units are functional units consisting of neurons, astrocytes and vasculature, in which the components are highly collaborative to maintain brain homeostasis and coordinate cerebral blood flow (CBF) (Eldahshan et al., 2019; Morrison and Filosa, 2019; Freitas-Andrade et al., 2020). Interactions among these cells produce at least three physiological significances. First, precisely coordinated neurovascular coupling (NVC) ensures stable hemodynamics to maintain normal glucose and oxygen supplies (Hillman, 2014; Lecrux and Hamel, 2016). Second, connections between astrocytes and vascular endothelial cells (ECs) construct the blood brain barrier (BBB) to regulate substance exchanges between the central nervous system (CNS) and the hematological system (Abbott et al., 2006). Third, neuronal activities, functions and metabolism can be subtly coordinated by astrocytes and vasculature (Perez-Alvarez et al., 2014; Freitas-Andrade et al., 2020). Ischemic stroke not only damages neurons, but also destroys the structural integrity of the vasculature, alters glial cell activity and the immune microenvironment. Stroke also destructs the integrity of BBB, a finely tuned structure composed astrocytes and ECs (Morrison and Filosa, 2019; Freitas-Andrade et al., 2020). Damages of BBB result in brain edema, leakage of inflammatory factors, infiltration of immune cells and hemorrhagic transformation (Wang and Parpura, 2016; Spronk et al., 2021).

Studies have demonstrated that neurons (Lang and McCullough, 2008), astrocytes (Chisholm and Sohrabji, 2016; Morrison and Filosa, 2019) and vasculatures (Memon and McCullough, 2018; Freitas-Andrade et al., 2020) respond differently to ischemic stroke between the sexes. The mechanisms that generate sexual dimorphism can be attributed to genetics, epigenetics, gonadal hormones and immunological differences. Sex chromosomes are related to ischemic stroke susceptibility, and genomic epigenetic modification affect ischemic injury responses (McCullough et al., 2016; Jiang et al., 2020). Gonadal hormone receptors are widely expressed in NVUs and thus modulate tolerance to brain ischemia (Yang et al., 2005; Ahmadpour and Grange-Messent, 2021; Sitruk-Ware et al., 2021). Immune cells including microglia (Kerr et al., 2019) and peripheral infiltrating immune cells (Ahnstedt and McCullough, 2019) also significantly affect NVUs between sexes. Additionally, a specialized immunological process, termed thromboinflammation, bridges thrombosis and neuroinflammation and can affect NVU stability under ischemic injury *via* crosstalk with the gonadal hormones (Roy-O’Reilly and McCullough, 2014; Rawish et al., 2020). Understanding these differences and their generating mechanisms may enable developing new sex-specific targets or drugs.

In this review, we summarize the differences in NVUs between the sexes and the mechanisms generating these dimorphisms. We first provide an overview of the differences in the neurons, astrocytes, and vasculatures and their crosstalk in response to stroke. Second, we discuss the genetic, epigenetic, endocrinal and immunological mechanisms that lead to these differences. These sex-specific differences in NVUs may provide a better understanding of ischemic stroke, reduce the gaps between laboratory and clinical research findings and enable developing more precise therapy for stroke using new sex-specific targets or drugs.

## Sexual Dimorphisms in Clinic

Table 1 summarizes the epidemiological and clinical features of stroke for both sexes. The incidence of stroke is much lower in premenopausal women than in age-matched men, but this trend reverses after menopause (Branyan and Sohrabji, 2020). In China, men under 65 years old have more strokes than do age-matched women, whereas in populations older than 65, women account for more stroke patients (Wang Y. J. et al., 2020). The risk factors for stroke differ between the sexes. Smoking and excessive drinking lead to more strokes in men, whereas diabetes mellitus, atrial fibrillation and migraines lead to more strokes among women. Sex-associated differences have also been evidenced in the clinical presentations of stroke. Unlike men, who primarily present typical symptoms such as hemiparesis, imbalance and dizziness, women present more atypical symptoms such as changes in mental status, aphasia, dysphagia, uroclepsia and visual disturbances (Labiche et al., 2002; Di Carlo et al., 2003; Roquer et al., 2003; Gargano et al., 2009). A recent study (Bonkhoff et al., 2021) found that zones near the posterior circulation in the left hemisphere are more vulnerable in women, which may partially account for these differences. Given these atypical symptoms, women are more likely to be misdiagnosed (Yu et al., 2019) and experience delays in receiving recanalization treatment (Boehme et al., 2017), thus leading to poorer outcomes. In the United States, women account for 60% of stroke-related deaths (Mozaffarian et al., 2015). However, researchers often enroll fewer female patients in stroke-related clinical trials (Strong et al., 2021), reflecting a universal ignorance of women. Therefore, the sex-associated differences in stroke and the sex-specific mechanisms affecting stroke processes must be studied to propose sex-specific diagnoses and treatment schemes.

**TABLE 1:** The epidemiological and clinical differences of stroke in two sexes.

	Male	Female	Reference
Incidence			Branyan and Sohrabji, 2020; Wang Y. J. et al., 2020
≤65 years	Higher	Lower	
>65 years	Lower	Higher	
Risk Factors	Male Specific: • Smoking • Excessive Drinking	Female Specific: • Diabetes Mellitus • Atrial Fibrillation • Migraine	Forster et al., 2009; Wang Y. J. et al., 2020
Clinical Presentations	More typical symptoms: • Hemiparesis • Imbalance • Dizziness	More atypical symptoms: • Coma • Aphasia • Dysphagia • Uroclepsia • Visual Disturbance	Labiche et al., 2002; Di Carlo et al., 2003; Roquer et al., 2003; Gargano et al., 2009
Mortality	40%	60%	Mozaffarian et al., 2015

## Sexual Dimorphism in Neurovascular Units During Ischemic Stroke

### General Responses of Neurovascular Units to Ischemic Injury

Ischemic stroke is an acute cerebral vascular accident caused by sudden obstruction of the cerebral arteries. Neuronal ischemia leads to oxygen and glucose deprivation and thus a reduction in adenosine triphosphate (ATP). ATP is the major source of energy for maintaining membrane electrical potentials, and a reduction in ATP can disrupt the electrical balance and further trigger excitotoxicity (Hinzman et al., 2015), featured with the release of glutamate and activation of N-methyl-D-aspartate receptors (NMDARs), α-amino-3-hydroxy-5-methyl-4-isoxazole-propionic acid receptors (AMPARs) and kainite receptors (KARs) (Zhou et al., 2018) to induce calcium influx. This Ca^2+^ overload leads to mitochondrial dysfunction and triggers the release of reactive oxygen species (ROS) (Szydlowska and Tymianski, 2010), ultimately resulting in neuronal death and neuroinflammation (Wieronska et al., 2021).

Astrocytes are double-edged swords in ischemic stroke. Astrocytes serve as power reservoirs and help neurons resist ischemic injury by storing glycogen (Gurer et al., 2009) and transferring mitochondria (Hayakawa et al., 2016). Astrocytes can also secrete neurotrophic factors (Pekny et al., 2016; Zhao et al., 2021) to alleviate neuronal death. However, hypoxic astrocytes become highly swollen and cannot clear glutamate (Menyhart et al., 2021), thus aggravate neuronal excitotoxicity. Moreover, brain ischemic leads to retraction of astrocytic endfeet from neurons and blood vessels, thus leading to structural disruption (Wang and Parpura, 2016). This retraction also impedes clearance of glutamate and results in aggravated excitotoxicity (Seidel et al., 2016). During ischemic stroke, glial retraction occurs in the infarct core, whereas in the penumbra area, astrocytic activation and gliosis, featured with upregulate glial fibrillary acidic protein (GFAP), occurs in parallel (Wang and Parpura, 2016). The activated astrocytes participate in neuroinflammation and polarize to the proinflammatory A1 phenotype or the anti-inflammatory A2 phenotype (Morrison and Filosa, 2019). In the late stages of stroke, activated astrocytes in the peri-infarct areas proliferate and form glial scarring in a process called astrogliosis (Yasuda et al., 2004). This glial scarring separates infarct areas from normal brain tissues (Wanner et al., 2013) and reduces inflammatory infiltration (Burda and Sofroniew, 2014). However, the scarring is weaker than the BBB and leaks inflammatory factors (Zbesko et al., 2018).

Ischemic injury to the vasculature can damage NVCs, break down the BBB and trigger thromboinflammation (Freitas-Andrade et al., 2020; Rawish et al., 2020). Endothelial nitric oxide synthase (eNOS) produces the vasodilator nitric oxide (NO) and thus physiologically modulates the CBF (Yamada et al., 2000). However, stroke may lead to eNOS dysfunction and NVC uncoupling (Freitas-Andrade et al., 2020). As the core of the BBB, ECs are attached *via* tight junction (TJ) proteins (Jiao et al., 2011). A breakdown of TJ increases BBB permeability (Jiao et al., 2011), promotes neuroinflammation and enhances the risk of hemorrhagic transformation (Spronk et al., 2021). Furthermore, vascular injuries induce thromboinflammation, featured with platelet activation and initiation of coagulation cascades, thus forming thrombi (Roy-O’Reilly and McCullough, 2014; Rawish et al., 2020), as well as interaction of activated platelets with neutrophils and lymphocytes, thereby inducing neuroinflammation (Duerschmied et al., 2013; Schuhmann et al., 2020).

Sex is an uncontrolled variable affecting neuronal death, astrocyte activation, glial scarring, BBB integrity, neuroinflammation and thrombosis. Sex-associated differences among the neurons, astrocytes, vasculature and their crosstalk during ischemic stroke are reviewed below and summarized in Table 2.

**TABLE 2:** Sex differences of NVU in ischemic stroke.

Cell	Sex Dimorphisms	Reference
Neuron	• Neuronal apoptosis in male neurons are independent of PARP-1. • Neuronal apoptosis in female neurons are associated with caspase cascades. • Estrogen suppresses neuronal autophagy in response to hypoxia or ischemia. • Loss of estrogen in aged females leads to more serious neuronal death and weakened neurogenesis	Du et al., 2004; McCullough et al., 2005; Szabo et al., 2006; Renolleau et al., 2007; Lang and McCullough, 2008; Li et al., 2017; Patrizz et al., 2021
Astrocyte	• Positive feedback production of estrogen is observed only in female astrocytes. • Female astrocytes have higher activity of P450, which catalyzes production of estrogen. • Estrogen enhances levels of GLT-1 and GLAST, who participate to clearance of glutamate. • Estrogen suppresses pro-inflammatory effects of astrocytes. • Estrogen reduces GFAP level and inhibits astrocytic activation. • Estrogen treatment to aged astrocytes exerts neurotoxicity effects because of reduced IFG-1.	Pawlak et al., 2005; Liu et al., 2007, 2008; Micevych et al., 2007; Arias et al., 2009; Lee et al., 2009; Kuo et al., 2010; Selvamani and Sohrabji, 2010; Chisholm and Sohrabji, 2016
Vessel	• Estrogen suppresses activity of NADPH-oxidase and reduces mitochondrial ROS production in endothelial cells. • Estrogen produces eNOS and PGI2 to dilate vessels, while testosterone generates TxA2 to constrict vessels. • Estrogen reduces BBB permeability. • Estrogen inhibits platelet activation and aggregation. • Estrogen replenish elevates risk of thrombosis by activating coagulating cascades in aged females.	Hayashi et al., 1995; Rubanyi et al., 2002; Gonzales et al., 2005; Liu et al., 2005; Krause et al., 2006; Miller et al., 2007; Duckles and Krause, 2011; Sohrabji et al., 2013; Roy-O’Reilly and McCullough, 2014

### Sex-Related Differences in Neurons

Laboratory studies, including *in vitro* models of oxygen and glucose deprivation (OGD) and its simulations and *in vivo* models such as middle cerebral artery occlusion (MCAO) models in adults and hypoxia-ischemia (HI) models in neonates, have revealed that females suffer less neuronal death at the cellular (Zhang et al., 2003), organic (Li et al., 2005), and systemic (Alkayed et al., 1998) levels.

Two types of apoptosis, intrinsic and extrinsic, have been reported. In the intrinsic pathway, mitochondria in injured cells release cytochrome C to form apoptosomes and further promote caspase cascade cleavage (Elmore, 2007). In the extrinsic pathway, oxidative stress in hypoxic neurons can damage DNA, activate poly-ADP ribose polymerase-1 (PARP-1) and release apoptosis-inducing factors (AIFs) (Susin et al., 2000). The intrinsic pathway plays a major role in female neuronal apoptosis, whereas the extrinsic pathway is more dominant in male neuronal apoptosis. Among cytosolic cytochrome C levels, females have more cleaved caspases in their neurons (Du et al., 2004). Caspase inhibition protects against ischemic stroke in only female, but not male rodents (Renolleau et al., 2007). Alternatively, PARP-1 and AIF levels are higher in males (Du et al., 2004). More interestingly, PARP-1 is detrimental to males but protective to females (McCullough et al., 2005). Genetic or pharmacological inhibition of PARP-1 protects against stroke in males, but has negative effects in females (McCullough et al., 2005; Szabo et al., 2006). These paradoxical sex-related differences in PARP-1 are partially related to estrogen (McCullough et al., 2005). In PARP-1-knockout mice, estrogen exerts harmful effects, but the exact mechanisms are unclear.

Hypoxia/ischemia also triggers autophagy, a cellular metabolic process in which autophagosomes with double-membrane structures engulf unwanted cytoplasmic proteins and damaged organelles to fuse with lysosomes and be lysed. Autophagy can lead to cell death but also maintain intracellular homeostasis (Wang et al., 2021), thus playing opposing roles in ischemic stroke. OGD (Patrizz et al., 2021) and starvation (Du et al., 2009) in the neurons of males show significantly more obvious autophagy than those of females, and a study by using neonatal HI models showed similar phenomena (Demarest et al., 2016). In MCAO-induced mouse models, pharmacological inhibition of autophagy reduces infarct volumes in male and spayed female mice but enhances these volumes in intact female mice (Patrizz et al., 2021). Estrogen inhibits autophagy in male (Patrizz et al., 2021) and ovariectomized female rodents (Li et al., 2017), exerting neuroprotective effects. Further inhibiting autophagy in female mice appears to induce deleterious effects.

Pyroptosis is a form of programmed cell death relating to inflammation, and is mediated by canonical inflammasome pathway, which depends on caspase-1, and non-canonical inflammasome pathway, which depends on caspase-4/5/11 (Man et al., 2017). It is reported that ischemic stroke enhances pyroptosis markers including caspase-1/11 (Gou et al., 2021), and inhibition of caspase-1 is neuroprotective (Ross et al., 2007). However, whether the progresses of pyroptosis differ in the two sexes remain unclear and need further exploration.

### Sex-Related Differences in Astrocytes

Sexual dimorphism in astrocytes under ischemic stroke is mainly influenced by gonadal hormones (Morrison and Filosa, 2019). Astrocytes in both sexes express sex hormone receptors. Astrocytes also serve as the major sources of steroid hormones in the CNS (Chisholm and Sohrabji, 2016), producing estradiol, progesterone and testosterone. Apart from the variances in hormonal levels between the sexes, interestingly, astrocytes in both sexes react differently to gonadal hormones. For example, estrogen or ER agonists can promote higher expressions of ERs and progesterone with a positive feedback mechanism, which is observed only in astrocytes in females (Micevych et al., 2007; Kuo et al., 2010). These structural and physiological characteristics of astrocytes lead to sexual differences in ischemic resistance, neuronal excitotoxicity, neuroinflammation and astrogliosis.

Astrocytes in females are more tolerant to OGD and H_2_O_2_-induced oxidative stress than are those of males, which can be partially attributed to higher activities of P450 and aromatase, enzymes to produce estradiol, in astrocytes of females (Liu et al., 2007, 2008). What’s more, glutamate transporter-1 (GLT-1) and glutamate-aspartate transporter (GLAST) are scavengers of glutamate expressed on astrocytes (Anderson and Swanson, 2000) and thus remove glutamate released by injured neurons to help alleviate excitotoxicity. Estrogen induces higher expressions of GLT-1 and GLAST (Pawlak et al., 2005; Lee et al., 2009), indicating that estrogen may reduce glutamate in ischemic brain tissues and further enhance tolerance to ischemic insult *via* astrocyte-neuronal crosstalk mechanisms.

Astrocytes participate in stroke-induced neuroinflammation, which can be regulated by sex hormones (Wang et al., 2014). Estrogen treatment polarizes astrocytes to the anti-inflammatory A2 phenotype (Chisholm and Sohrabji, 2016). OGD in astrocytes leads to mitochondrial dysfunction and release of ROS, the promoters of inflammation; estrogen can suppress this process (Guo et al., 2012). Lipopolysaccharide (LPS) triggers inflammation in cells and animal models. Estrogen strongly impedes LPS-induced increases in IL-1β, TNFα, MMP-9, IL-6, IP-10, and transcription of NF-κB in astrocytes (Azcoitia et al., 2010; Chisholm and Sohrabji, 2016). The immunomodulatory effect of estrogen on astrocytes helps reduce injuries in premenopausal females.

Previous reports suggest that female-associated hormones, including estrogen (Perez-Alvarez et al., 2012) and progesterone (Djebaili et al., 2005), inhibit astrocytic activation. GFAP levels are lower in female mice and are fluctuant independent of the estrus cycle (Arias et al., 2009). Estrogen increases expression of the proliferation-inhibiting gene, N-myc downstream-regulated gene 2 (Ndrg2), in astrocytes (Ma et al., 2014). These findings suggest that estrogen may inhibit astrogliosis after stroke, which was verified in a recent study (Wang J. et al., 2020).

### Sex-Related Differences in the Vasculature

Sexually dimorphic variances in infarct volumes also depend on the different reactions of the vasculature to stroke (Sohrabji et al., 2013; Freitas-Andrade et al., 2020). Reproductive hormones play key roles in vasculature diversity (Rubanyi et al., 2002; Memon and McCullough, 2018). Hormones modulate cellular viability, regulate vascular diameters and adjust the tightness of cellular junctions in cerebral vessels.

Estrogen suppresses NADPH-oxidase (Miller et al., 2007) and mitochondrial ROS production (Duckles and Krause, 2011) in ECs, enhances levels and activities of enzymes related to the respiratory chain and tricarboxylic acid cycle (Stirone et al., 2005a,b), maintains stable energetic metabolism and reduces EC apoptosis. Estrogen promotes eNOS activity to produce higher NO levels (Hayashi et al., 1995) and enhances prostacyclin (PGI2) levels (Rubanyi et al., 2002; Krause et al., 2006), resulting in blood vessel dilation. On the other hand, testosterone increases thromboxane (TxA2) activity and leads to vasoconstriction (Gonzales et al., 2005). These findings suggest that reduced CBF is less severe in younger female stroke patients and animals than in age-matched males. Estrogen also reduces BBB permeability (Liu et al., 2005) in female rodents and lowers peripheral infiltration (Sohrabji et al., 2013). Vessels exhibiting ischemic injury trigger thromboinflammation, which exhibits sexual dimorphism (Roy-O’Reilly and McCullough, 2014). The influences of thromboinflammation and its sex-associated differences are detailed below.

### Effects of Age on Neurovascular Units Between the Sexes

Although younger female rodents show lower infarct volumes and less severe neurological impairments than do age-matched males, these trends are reversed in older rodents (Manwani et al., 2011). These findings are consistent with clinical reports that postmenopausal women experience a greater incidence and severity of stroke (Towfighi et al., 2007). Hence, age influences the diverse responses to stroke between males and females.

Without the protective effects of female hormones, neurons are more vulnerable to ischemic/hypoxic injury and have weaker neurogenesis (Lang and McCullough, 2008; De Nicola et al., 2018). Furthermore, loss of estrogen enhances astrocytic GFAP, promotes gliosis and elevates proinflammatory A1 astrocytes (Morrison and Filosa, 2019; Moulson et al., 2021). Estrogen withdrawal can also diminish eNOS activity, prevent vascular dilation, and enhance BBB permeability in injured brains (Roy-O’Reilly and McCullough, 2014; Memon and McCullough, 2018). Notably, in perimenopausal women and middle-aged female rodents, reduced estrogen and progesterone and elevated follicle-stimulating hormone (FSH) and luteinizing hormone (LH) increase cholesterol levels and stroke risk (Sohrabji et al., 2013; McCarthy and Raval, 2020).

Conversely, replenishing ovarian hormones negatively affects older female rodents (Selvamani and Sohrabji, 2010; Leon et al., 2012) and patients (Carrasquilla et al., 2017). Estrogen treatment in older females exerts neurotoxic effects, which is related to loss of trophic factor IGF-1 (Selvamani and Sohrabji, 2010). Different administration modes and dosages of estrogen may influence estrogen’s immunomodulatory effects on ischemic stroke (Strom et al., 2011; Shepherd et al., 2020), as detailed below. Replenishing estrogen also elevates the risk of thrombosis by activating coagulating cascades in aged females (Roy-O’Reilly and McCullough, 2014; Laliberte et al., 2018).

Apart from hormonal effects, the X chromosome seems to play more marked roles in older females (Traylor et al., 2015), and the exact mechanisms are elucidated below. Generally, the roles of age in both sexes remain poorly explored. Appropriate therapies are urgently needed for older stroke patients, especially older women. However, young male rodent models of stroke are used more widely in laboratory research (Spychala et al., 2017; Bushnell et al., 2018), which is somewhat inadequate for meeting these clinical needs.

## Mechanisms for Generating Sexual Dimorphism

**FIGURE 1:**
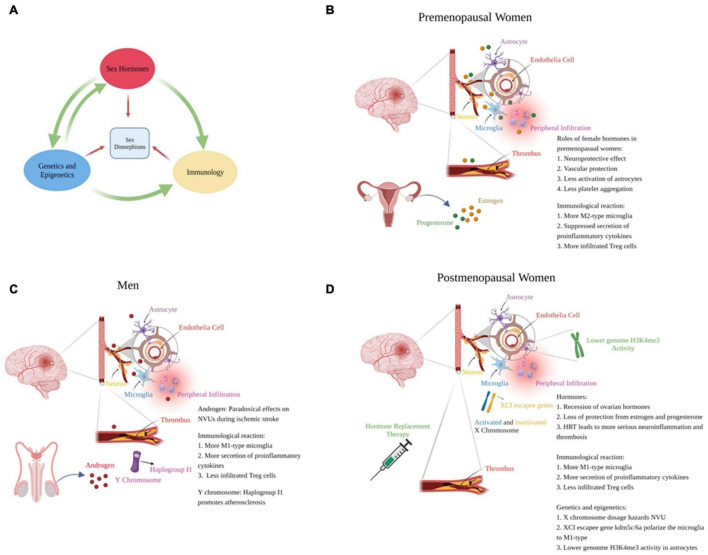
Mechanisms to generate sex-specific responses to ischemic stroke. **(A)** Sex hormones, genetics and epigenetics and immunological reactions promote different responses to stroke dependently or collaboratively between the sexes. **(B–D)** respectively represent the responses of NVUs to ischemic stroke in premenopausal women, men and postmenopausal women (created with BioRender.com).

Gonadal hormones play major roles in generating sexual dimorphism in ischemic stroke. Hormones directly affect NVU functions and modulate genetic expression *via* nuclear receptors, thus regulate cellular death, NVU crosstalk and inflammation. Different genetic expression profiles between sexes also leads to variant NVU and immunological responses. Herein, we summarize the hormone-gene-immune crosstalk that lead to different reactions to stroke in both sexes (Figure 1).

### Hormones

#### Estrogen

The CNS and the immune system in both sexes are reactive to estrogen (Xu et al., 2021). Studies suggest that estrogen exerts protective effects in cerebral injuries by suppressing oxidative stress and reducing cellular apoptosis (Behl et al., 1997). Estrogen also activates anti-apoptotic PI3K/AKT (Mannella and Brinton, 2006) and MAPK/Erk (Wang et al., 2011) pathways and inhibits the pro-apoptotic JNK pathway 2 (Yao et al., 2007), ultimately reducing apoptosis. Estrogen regulates transcription of neurotrophic factors such as such as IGF-1, BDNF, GDNF, and VEGF (Campos et al., 2012; Pietranera et al., 2015) and suppresses neuroinflammation by inhibiting NF-κB transcription activities (Cook et al., 2018) and enhancing STAT3 (Sehara et al., 2013) and PPARγ (Kinouchi et al., 2012) transcription activities.

Interestingly, toxic effects have also been reported. The possible mechanisms include exacerbation of neuroinflammation (Xu et al., 2004) and overactivation of blood coagulation (Roy-O’Reilly and McCullough, 2014). Estrogen also paradoxically exerts proinflammatory effects by releasing cytokines and promoting leukocyte infiltration in brain-injury models including MCAO models (Xu et al., 2004). Estrogen is also reported to enhance levels of coagulant factors and promote thrombosis (Roy-O’Reilly and McCullough, 2014). Diabetes (Xu et al., 2004) and aging (Rossouw et al., 2002) may contribute to more negative effects from estrogen. Moreover, oral administration of estrogen leads to a greater chance of thrombosis. First-pass elimination of oral contraceptives stimulates hepatocytes to synthesize coagulatory factors, resulting in hypercoagulation (Dupuis et al., 2019). Furthermore, high-dosage of estrogen is likely to promote inflammation (Shepherd et al., 2020).

#### Progesterone

Progesterone is another female-associated neuroprotective hormone recognized progesterone receptors. Progesterone reduces infarct volumes, alleviates neurological deficits and improves stroke outcomes in animal models of stroke (Wong et al., 2013; Zhu et al., 2017). Progesterone regulates GABA_A_ receptor functions, indicating that progesterone can antagonize excitotoxicity (Hosie et al., 2006; Zhu et al., 2017). Progesterone can also reduce oxidative damage, cellular apoptosis, BBB destruction and hemorrhagic transformation (Guennoun, 2020). Moreover, similar to estrogen, progesterone alleviates inflammation and modulates microglial and astrocytic activities (Djebaili et al., 2005; Won et al., 2015; Aryanpour et al., 2017). Taken together, progesterone collaborates with estrogen to protect against cerebral injury in reproductive females.

#### Testosterone

In the CNS, testosterone recognizes androgen receptor to mediate genetic transcription or regulate intracytoplasmic signaling pathways to influence cellular apoptosis, BBB integrity, CBF and neuroinflammation (Ahmadpour and Grange-Messent, 2021). Roles of male hormones in stroke are ambiguous and poorly studied, which requires further research. Compared with those of age-matched women, higher testosterone levels in younger men are related to a higher risk of stroke (Wang et al., 2013). However, decreased testosterone in aged men increases the possibility of stroke (Chock et al., 2012). Testosterone showed both neurotoxic (Cheng et al., 2007) and neuroprotective (Cheng et al., 2011) effects in MCAO-induced rodent models. These different effects are partially related to testosterone dosage, as low-dose androgen is reportedly protective, whereas high-dose androgen is toxic (Uchida et al., 2009). Additionally, testosterone can be turned into estrogen by aromatase (Roselli et al., 2009), thus generating protective effects. In general, roles of testosterone in stroke are controversial and needs further researches.

#### Oxytocin

Steroid hormones cannot sufficiently explain that women at reproductive age have lower incidence and less severity of ischemic stroke, other factors, particularly oxytocin, should be taken into account. Oxytocin is a neuropeptide that promotes lactation and parturition. Apart from mammary gland and uterine, oxytocin receptors are widespread in the CNS and immune system (Wang et al., 2015). The levels of oxytocin are higher in women than in men (Kunitake et al., 2021). It is reported that oxytocin mediates neuroprotective effects in ischemic stroke (Karelina et al., 2011). The possible mechanism can be attributed to its crosstalk with neuroimmune and relieving inflammation (Wang et al., 2015). The reduction of oxytocin at aged women may account for the exceptional high incidence of ischemic stroke in postmenopausal women.

### Genetics and Epigenetics

Although sex hormones play major roles in generating sex differences, their diverse functions in the CNS reveal that hormonal mechanisms cannot sufficiently explain the pathophysiological differences in stroke between the sexes. Studies suggest that heritability of stroke is significantly higher in women than in men (Touze and Rothwell, 2008; Traylor et al., 2015). Women with stroke are more likely than are men to have maternal stroke histories and to have affected sisters but unaffected brothers (Touze and Rothwell, 2007). Furthermore, genetics have stronger effects on older women who are stroke patients than on age-matched men (Traylor et al., 2015). Thus, sexual dimorphisms in genomic expression profiles are another important source of sexual differences. The innate expressive profiles and activities of the sex chromosomes modulate sex-specific responses to ischemic stroke. Genetic expressions on autosomes also differ between the sexes. Substantial differences in epigenetic modifications of genomes and histones also lead to sex differences in stroke. Herein, we summarize the roles of genetic and epigenetic influences in neurovascular functions of both sexes.

#### X Chromosome

Different X-chromosome dosages contribute to varied responses to stroke, which are more pronounced in older patients. Mice with XX complement have higher levels of pro-atherogenic lipids and greater risk of atherosclerosis than do mice with XY complement (AlSiraj et al., 2019). A study using four-core-genotype (FCG) mice (XX males or females, and XY males or females, whose testis-determinant gene *Sry* was spliced from the Y chromosome to autosome 3) found that both aged XX males and females showed higher infarct volumes, and XX complement leads to higher levels of proinflammatory cytokines in aged mice (McCullough et al., 2016). To balance the X-chromosome gene dosage, XCI (X-chromosome inactivation) randomly devitalizes one X chromosome during development (Nguyen and Disteche, 2006). Notably, 15–25% of genes on the X chromosome can escape XCI (Carrel and Willard, 2005), resulting in mosaicism in X-chromosome gene expression in females and leading to differences in immunological responses (Florijn et al., 2018). Especially, XCI escapee genes, *kdm5c* and *kdm6a*, demethylate H3K4me3 and H3K27me3, respectively, and further epigeneticly modify interferon regulatory factor (IRF4/5) expression, thus result in proinflammatory alteration of the microglia in females (Qi et al., 2021).

Genetic expressions of the X chromosome in ischemic stroke differ between the sexes. Female-specific X-expressing genes include TIMP1, DDX3X, IKBKG, PRKX, GLA, MAOA/B, and MAGE families, which are related to post-translational modification, small-molecule biochemistry and cell-cell signaling (Stamova et al., 2012). Male-specific genes include EFNB1, CYSLTR1, IGBP1, and TLR-7, which are associated with cellular movement, development, trafficking and death (Stamova et al., 2012). These genes exert both protective and detrimental effects on NVUs and can serve as potential sex-specific biomarkers and therapeutic targets.

#### Y Chromosome

The Y chromosome generates male-specific reactions to stroke. Eales et al. reported that *Haplogroup I1* in the male-specific region of the Y chromosome was associated with an increased risk of atherosclerosis (Eales et al., 2019). After stroke in men, genes on Y chromosome, including VAMP7, CSF2RA, SPRY3, DHRSX and PLCXD1, EIF1AY and DDX3Y, are differentially expressed compared with those of healthy men. These differentially expressed genes on the Y chromosome are related to mechanisms regulating immunology, RNA metabolism, vesicle fusion and angiogenesis (Tian et al., 2012b).

#### Whole-Genome Differences

Microarray analysis of the whole genome in blood samples from healthy persons and stroke patients of both sexes revealed sex-specific expression profiles. In elderly stroke patients, female-specific genes are associated with p53, high-mobility group box-1, HIF-1α, IL-1, IL-6, IL-12, IL-18, acute-phase response, Th cell, macrophage, and estrogen signaling, whereas male-specific genes are related to integrin, integrin-like kinase, actin, TJ, Wnt/β-catenin, RhoA, fibroblast growth factors, granzyme, and TNFR2 signaling (Tian et al., 2012a). Another study analyzed samples from older patients with cardioembolic stroke and found that female genes were associated with cell death and survival, cell-cell signaling and inflammation, whereas male genes were related to cellular assembly, organization and compromise (Stamova et al., 2014).

Epigenetic modifications are also sex-specific during ischemic stroke (Spychala et al., 2017). Histone methylation, one type of epigenetic regulation, is related to T-lymphocyte functions (He et al., 2013), astrocyte activities (Chisholm et al., 2015) and oxidative stress (Zhao et al., 2016). Among which, the roles of histone-3 lysine-4 trimethylation (H3K4me3) are well-studied in both sexes. Female mouse brains exhibited larger peak amounts of H3K4me3, and the genes and loci with increased H3K4me3 were related to synaptic functions (Shen et al., 2015). In a MCAO-induced mouse model, H3K4me3 activity in astrocytes from older females was decreased compared with that in younger females, leading to decreased VEGF levels and higher cellular apoptosis (Chisholm et al., 2015). These studies suggest that H3K4me3 can serve as a female-specific epigenetic marker following ischemic stroke in aged female patients.

### Neuroinflammation and Immunological Responses

Sex differences in inflammatory responses also cause variable sex-specific reactions to stroke (Spychala et al., 2017) independently or by collaborating with gonadal hormones, genetic expression and epigenetic modification. Herein, we review sexually dimorphic inflammatory responses and their crosstalk with NVUs during stroke.

#### Microglia

Microglia are *in situ* immune cells in the CNS and serve as first-line defenses for maintaining NVU homeostasis. Pathological stimulation, such as brain ischemia, activates the microglia and the activated microglia can polarize to the pro-inflammatory M1 and anti-inflammatory M2 phenotype (Kanazawa et al., 2017). Upon ischemic stroke, microglia can be mainly polarized to M2-type at the ultra-early stage (Hu et al., 2012). A few days later, the M1-type become more dominant, especially in the peri-infarct area (Hu et al., 2012), ultimately lead to BBB destruction and infiltration of peripheral immune cells (Anrather and Iadecola, 2016).

There are sex differences in microglia during ischemic stroke (Eldahshan et al., 2019). Microglia express gonadal hormone receptors, indicating that sex hormones can regulate microglial reactivity and phenotypes (Kerr et al., 2019). Interestingly, transplantation of microglia from female to male brains reduced infarct volume, suggesting that the intrinsic anti-inflammatory effects of female microglia are independent of hormones (Villa et al., 2018). Apart from the influences of gonadal hormones, innate differences between male-derived and female-derived microglia on genetic and proteomic levels are reported (Guneykaya et al., 2018; Villa et al., 2018).

Males have a higher antigen-presenting capacity and are easier to respond to stimuli (Guneykaya et al., 2018), while female microglia are more neuroprotective (Villa et al., 2018). During ischemic stroke, microglia in females exert higher levels of the M2 phenotype, whereas microglia in males exhibit more of the M1 phenotype (Villa et al., 2018; Kerr et al., 2019). Microglia in females are more sensitive to IL-4 and IL-10 and have higher expressions of the anti-inflammatory markers, IL-4 and CD206, in ischemic brains (Bodhankar et al., 2015; Seifert et al., 2017). Microglia in males express higher levels of Iba1, TLR2 and TLR4, thus showing higher activity and a proinflammatory phenotype (Spychala et al., 2017; Villa et al., 2018; Kerr et al., 2019). However, microglia in aged stroke patients may show completely opposite phenotypes. Microglia from aged females produce higher levels of TNF-α, IL-1β, CXCL10, and kdm5c/6a (Kerr et al., 2019; Qi et al., 2021), thereby exhibiting pro-inflammatory effects.

#### Peripheral Immune Cells

Ischemic stroke triggers infiltration of neutrophils, monocytes/macrophages, lymphocytes and other immune cells (Jiang et al., 2020). These inflammatory cells further influence NVU functions, which react differently between the sexes.

Physiological levels of both testosterone and estrogen can suppress LPS-induced TNF-α release in rodent monocytes/macrophages (Shepherd et al., 2020). However, another study reported contradictory results, showing that ovariectomized mice had reduced levels of proinflammatory cytokines such as TNF-α, IL-6, and TLR4 in their monocytes/macrophages (Rettew et al., 2009). These results further suggest a dual role of estrogen in inflammation. For neutrophils, estrogen can suppress the formation of neutrophil extracellular traps and attenuate expression of proinflammatory cytokines in females (Miller et al., 2004; Kaplan and Radic, 2012). Sex-specific roles of lymphocytes, especially T cells, are also widely documented. Males have more infiltrating CD3^+^ T cells (Brait et al., 2010), more IL-4-regulated cerebral infiltration of CD4^+^ and CD8^+^ T cells (Xiong et al., 2015). Regulatory T (Treg) cells are beneficial during ischemic stroke, and younger females have more Treg cells during stroke (Dotson et al., 2015). Age may influence immunological activities between the sexes. In middle-aged female mice, macrophages exert proinflammatory effects by releasing more IL-1β and IL-6 (Curuvija et al., 2017). Furthermore, in older patients and animals, females have fewer Treg cells after brain ischemia (Yan et al., 2012; Ahnstedt et al., 2018).

#### Thromboinflammation

As a specialized immunological response, thromboinflammation bridges thrombosis and neuroinflammation, and platelets play a central role (Rawish et al., 2020). Injured vasculature attracts platelet adhesion and aggregation, and the latter directly interacts with neutrophils and monocytes (EdRainger et al., 2015). T cells exhibit harmful effects during stroke by interacting with platelets (Li et al., 2006; Hu et al., 2010). Furthermore, platelets induce neuronal apoptosis due to Fas ligand release (Schleicher et al., 2015).

Sex hormones receptors are also located on platelets (Roy-O’Reilly and McCullough, 2014; Dupuis et al., 2019), indicating that thromboinflammation is sexually dimorphic. Roles of sex hormones in platelets are controversial. *In vivo* studies suggest that in young rodents, testosterone promotes thrombosis, while estrogen attenuates the pro-thrombotic state (Emms and Lewis, 1985). Interestingly, however, patients receiving HRT show higher risk of cardiovascular diseases (Rossouw et al., 2002), suggesting that aging may be a potential factor that reverses the antithrombotic effects of estrogen. Other evidence suggests that coagulant factor thrombin leads to more severe estrogen-dependent ventricular dilation and white matter damage in females (Peng et al., 2021), suggesting that estrogen stimulates thrombotic damage. In addition, oral contraceptives can overactivate blood coagulation cascades *via* hepatic first-pass elimination (Dupuis et al., 2019). Thus, estrogen plays ambiguous roles in thrombosis and its subsequent inflammation, which requires further investigation.

In postmenopausal women without estrogen, thrombosis leads to severer damage during stroke. A recent meta-analysis suggested that older female stroke patients showed higher levels of coagulant factors (van der Weerd et al., 2021). t-PA treatment is reported to be less effective in women (Manwani and McCullough, 2019; Sheth et al., 2019; Yu et al., 2019). Women with atrial fibrillation are less sensitive to warfarin and have a higher risk of stroke (Sullivan et al., 2012). Thrombosis in older women remains poorly understand, and research on antithrombotic therapies should focus more on this population.

## Prospects and Current Limitations

It is comforting that more and more researches have paid attention sex differences in stroke, especially to women. Opinions to prevention and treatment of women stroke patients have been proposed (Bushnell et al., 2014; Pezzella et al., 2014). Although the delay of diagnosis and treatment are more possible in women and women are less likely to receive recanalization therapy as mentioned above, women treated with rt-PA may have higher recanalization rate (Savitz et al., 2005). The possible mechanism is that thrombi in women have richer fibrin and less platelet, which are easier to be dissolved (Forster et al., 2009). A report from Women Stroke Association suggests that aspirin decreases the risk of ischemic stroke by 24% in women (Ridker et al., 2005). However, use of aspirin is lower in women (Glader et al., 2003). Menstruation influences stroke phenomenology, and irregular menstrual cycle, early menarche and premature menopause are positively related to stroke risk (Roeder and Leira, 2021).

Several sex-specific neuroprotective drugs have been implicated in clinical trials. For example, uric acid is targeted toward women (Llull et al., 2015), while minocycline is effective for men (Honarpisheh and McCullough, 2019). Laboratory studies have also reported new sex-specific drugs and potential therapeutic targets. Pharmacological blockade of Na-K-Cl cotransporter 1 is more protective in male mice (Huang et al., 2019). Additionally, programmed death ligand 2 neutralization (Seifert et al., 2019) is specifically protective in males. For females, blood vitronectin specifically induces detrimental outcomes (Jia et al., 2020). Furthermore, deficiency of endothelial SIRT3 leads to diastolic dysfunction in aged females (Zeng et al., 2020), indicating a potential female-specific target for preserving NVC after brain ischemia.

Several limitations exist in the current clinical and basic research, which restrict our understanding of the pathophysiological processes and laboratory-clinical translation of stroke:

1. Female animals are often underused or lacking in laboratory studies, and women are less often recruited and analyzed in clinical trials. Because stroke is sexually dimorphic, both sexes should be examined equally in stroke research in this era of precision medicine.
2. Age differentially influences reactions to stroke between the sexes. Both clinical and laboratory data reveal poorer outcomes in older women and animals, but basic studies targeting these populations are lacking.
3. The failed clinical translation of HRT in postmenopausal women with stroke suggests that studies regarding the effects of gonadal hormones on stroke are biased. Although estrogen exhibits neuroprotective effects during stroke, adverse effects of estrogen are also reported. Appropriate dosages, delivery pathways, and administrative duration of estrogen and their different roles in younger and older females should be finely studied in future works.
4. The contradictory roles of testosterone in males suggest that proper intervention with male hormones may benefit men.
5. Hormone-independent sex differences are poorly understood and minimally studied. FCG mice are convenient and practical for researching functions of sex chromosomes and hormone-gene interactions. Neuroinflammation in stroke is widely documented, but documentation of sexual differences is lacking. Adequate understanding of hormone-gene-immune networks between the sexes may reveal more accurate targets for treating stroke.
6. Sex-associated differences in thromboinflammation are ambiguous and poorly understood. Clinical treatments for ischemic stroke focus mainly on the contradiction between thrombosis and thrombolysis. Sex-associated differences in thromboinflammation and use of anticoagulants and thrombolytic drugs should be considered in future research.

## Conclusion

**FIGURE 2:**
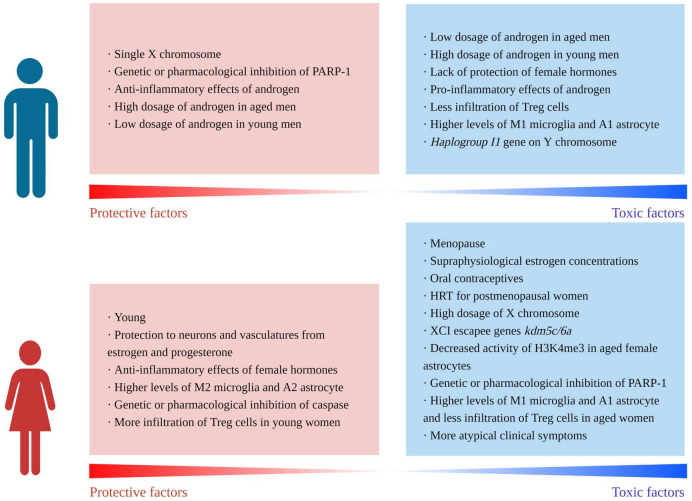
The protective and toxic factors for the two sexes during ischemic stroke (created with BioRender.com).

Stroke is a common cerebrovascular disease with obvious sexual dimorphism. Figure 2 summarizes the differences in sex-specific protective and toxic factors. Clinically, young women have a lower incidence and better outcomes from stroke compared with those of age-matched men; however, this trend reverses after menopause. Experimental data from rodents reveal similar consequences. Neuronal apoptosis pathways in both sexes show distinct and innate differences in that male neurons are PARP-dependent, whereas female neurons are caspase-dependent. Other differences in NVUs are modified by hormone-gene-immune networks, which can be altered by age. Premenopausal females are protected by estrogen and progesterone and thus exhibit less neuronal death, restricted astrocyte activation, less BBB leakage, higher CBF and less neuroinflammation. However, aging leads to higher expressions of toxic genes and pro-inflammatory alterations of immune profiles in females, leading to poorer NVU crosstalk and severer neuroinflammation. Additionally, exogenous administration of estrogen leads to deteriorated stroke outcomes in older females. Future studies should focus more on sexual dimorphism in stroke. Therapies for older women are urgently required. Roles of gonadal hormones, especially their adverse effects, should be considered. Use of FCG mice may help better understand the sexual dimorphism of stroke.

## Author Contributions

TT proposed the idea and drafted the manuscript. LH assisted literature retrieval and filtration, and helped manuscript revision. YL drew the illustrations and assisted manuscript polish. XF provided the copyright of Biorender. FY and JL were responsible for supervising. SC and GC provided financial and executive supports. All authors contributed to the article and approved the submitted version.

## Conflict of Interest

The authors declare that the research was conducted in the absence of any commercial or financial relationships that could be construed as a potential conflict of interest.

## Publisher’s Note

All claims expressed in this article are solely those of the authors and do not necessarily represent those of their affiliated organizations, or those of the publisher, the editors and the reviewers. Any product that may be evaluated in this article, or claim that may be made by its manufacturer, is not guaranteed or endorsed by the publisher.
